# CT-scan examination of sacred Vodun objects (Benin): between diagnosis and research ethics

**DOI:** 10.1016/j.redii.2026.100065

**Published:** 2026-09-04

**Authors:** Philippe Charlier, Didier N’Dah, Isabelle Huynh, Constant Glele, Franck Hounlelou, Vincent Hounlelou, Luc Brun, Dodji Amouzouvi

**Affiliations:** a Laboratory Anthropology, Archaeology, Biology (LAAB), UFR Simone-Veil – santé, Université de Versailles-Saint-Quentin-en-Yvelines (UVSQ), 2 avenue de la Source-de-la-Bièvre, 78180, Montigny-Le-Bretonneux, France; b Department of Epidemiology and Public Health, Hôpital Raymond-Poincaré (AP-HP), 104 boulevard Raymond-Poincaré, 92380, Garches, France; c Laboratoire d’arts, d’archéologie, de cultures et d’expertise patrimoniale et touristique (Laacept), Université d’Abomey-Calavi, Abomey-Calavi, Benin; d Service d’imagerie des urgences et spécialisée, Hôpital universitaire Pitié-Salpétrière (AP-HP), 83, boulevard de l’Hôpital, 75013, Paris, France; e Various Vodun sanctuaries, Abomey, Benin; f Department of Pathology, University Hospital, Parakou, Benin; g Laboratoire d’analyse et de recherche religions espaces et développement (Larred), Université d’Abomey-Calavi, Abomey-Calavi, Benin

**Keywords:** CT-scan, Archaeology, Anthropology, Research

## 1 Introduction

Increasingly, radiological technology has found its place beyond the field of healthcare. In archaeology, it has become an indispensable technological tool for gaining a better understanding of artifacts from ancient contexts and the internal structure of works of art [1]. Medical scanning and radiology have established themselves as essential prerequisites to laboratory excavations or physical restoration [2]. Radiologists and radiology technicians are increasingly being trained in the acquisition and interpretation of such data [3].

The specific added value of the present study lies not simply in the use of computed tomography (CT) imaging for archaeological artifacts (which has already been widely demonstrated) but in its application to religious *vodun ritual objects whose integrity could not ethically be altered*. Unlike most previous CT studies focused on mummies, funerary assemblages, sculptures, or conservation issues, this work combines three original dimensions: (1) the investigation of living religious objects that remain culturally and spiritually active for descendant communities; (2) the explicit integration of community-based ethical considerations into the imaging protocol, where CT-scan is interpreted as a “medical examination” rather than a destructive intervention; and (3) the identification of internal ritual deposits and sacrificial residues (fatty libations, vegetal offerings, embedded votive objects) that are inaccessible through conventional archaeological approaches without desacralization or physical opening. This short study therefore proposes CT imaging not only as a technical tool for non-destructive analysis, but also as a culturally acceptable methodology for the investigation of sacred heritage. In this sense, it expands previous archaeological CT applications toward a new interdisciplinary field at the intersection of radiology, heritage ethics, ethnography, and the anthropology of living ritual practices.

## 2 Material and method

**Fig. 1:**
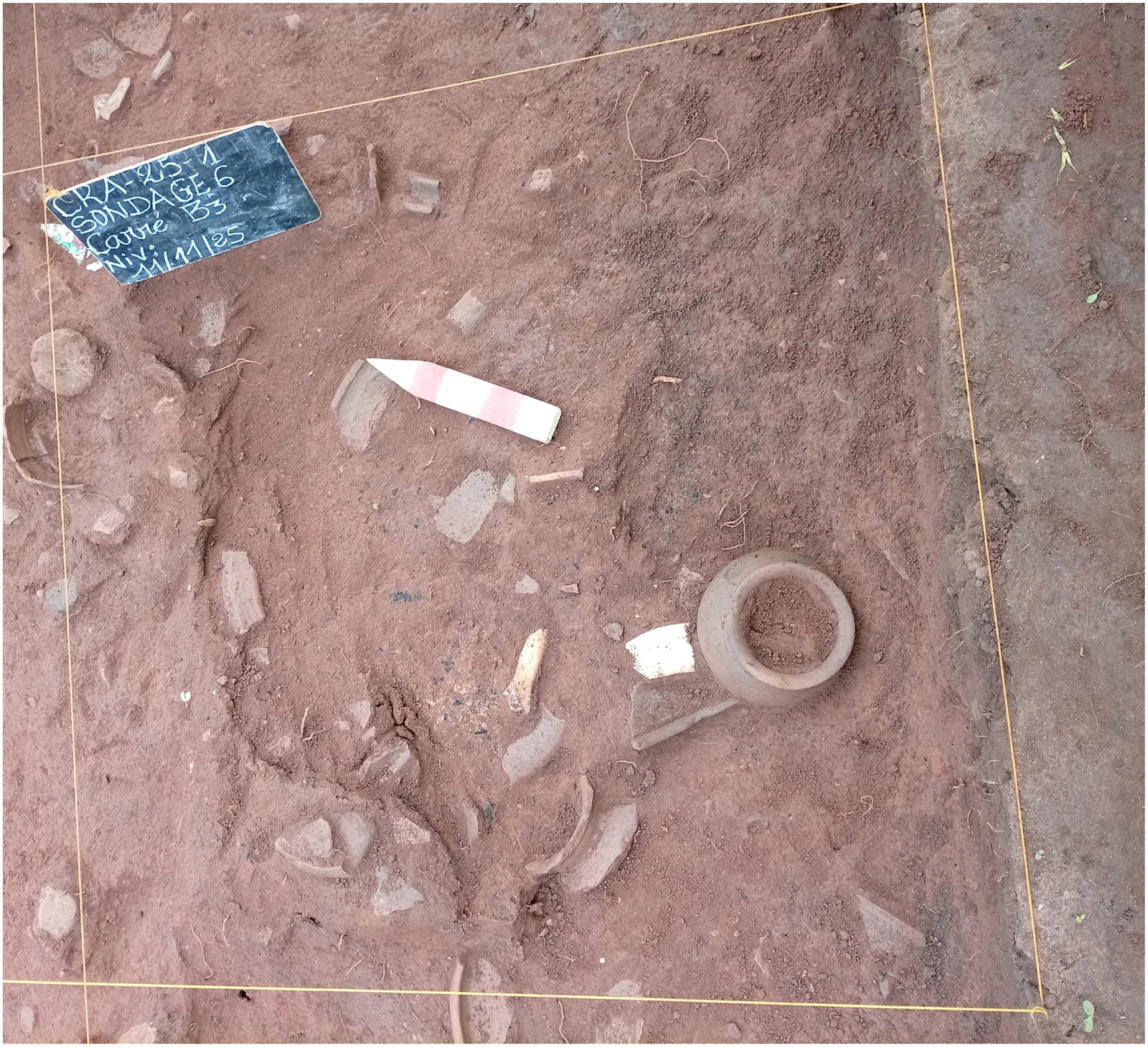
CT-scan examination of sacred Vodun objects (Benin): In-situ aspect of the ceramics discovered in the archaeological excavations at Abomey.

In this particular case, two ceramic pieces (#1 and #2) of respectively 13 and 20 cm major axis, from archaeological excavations within the royal palaces of Abomey (Benin) (Fig. 1), as part of an international AROMA mission funded by the French Ministry for Europe and Foreign Affairs, and a ritual calabash (#3) of 40 cm major axis collected at the Vodun material market in Bohicon (Benin) and now part of the Laboratory Anthropology Archaeology Biology (LAAB) collection underwent “virtual autopsies” using a CT-scan examination.

Technical parameters were: CT-scanner without injection of contrast medium (thin sections with subsequent three-dimensional [3D] reconstruction: Siemens Somatom Definition Edge, slice thickness 0.60 mm; tube voltage 120–140 kV; dose per section 35 mAs; final DLP of 272.73 mGy/cm; FOV 320 mm; and filter sharp yb detail bone.

## 3 Results

The use of this tool proved particularly judicious given the ritual necessity of not desacralizing each object, that is, of preserving their physical integrity, in strict accordance with the Vodun community from which they originate. But we were then facing two limits: first, did we have empirically a homogenous Vodun community, and if so, who are the members and who decided the membership? Second, can we really establish the origin of these objects when we know that most of them circulate a lot in space and time?

In this context, sacred and ritual objects are the repositories of a spirituel power, a kind of vessel for a divine spirit (*vodun* in the Fon language) that is ultimately one with it [4]. In this zoomorphic (or anthropomorphic) view, altering their integrity would therefore be to mutilate these sacred beings (as during an autopsy or dissection), while scanning the object logically amounts to subjecting it to a medical examination (a diagnosis, in this case) with a much less (but still persistent) risk of desacralization [5].

**Fig. 2:**
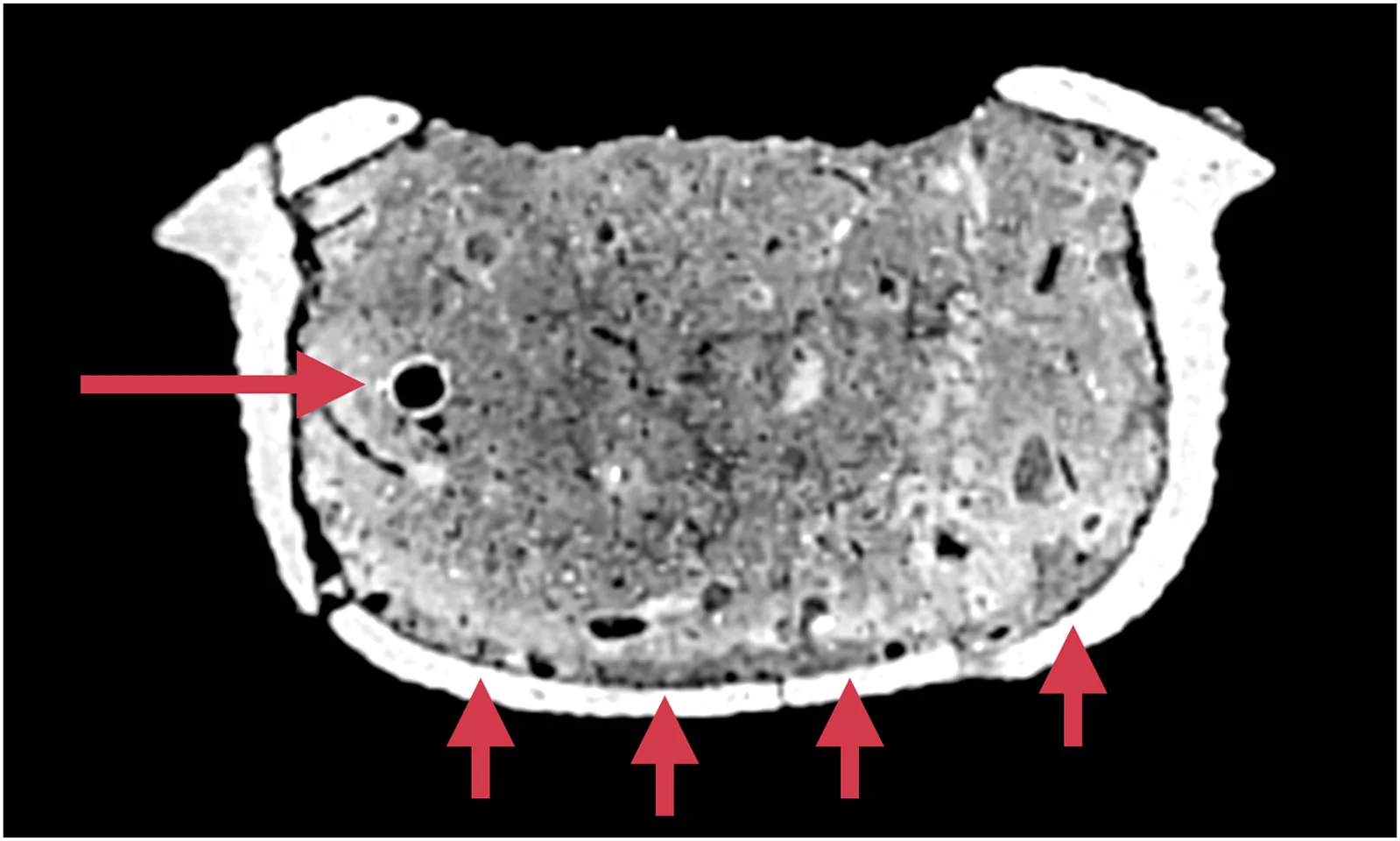
CT-scan examination of sacred Vodun objects (Benin): Cross-vertical-section of ceramic #1 with a negative imprint of a plant offering (peanut?) (large arrow) and a low-density slope (palm oil imbibition?) (small arrows). In addition, the profile and thickness of the entire ceramic, as well as its areas of weakness, appear and are measurable.

**Fig. 3:**
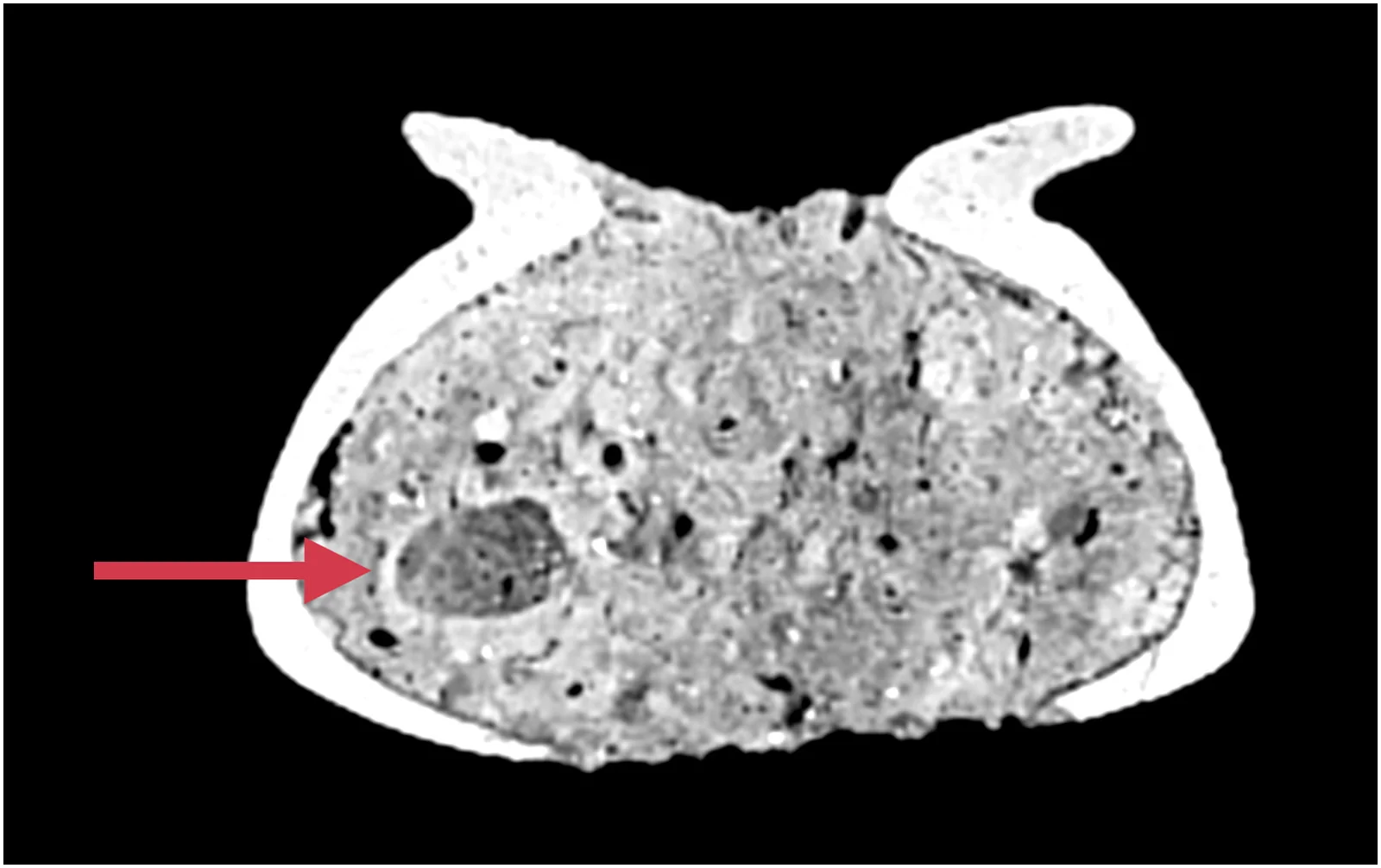
CT-scan examination of sacred Vodun objects (Benin): Cross-vertical-section of ceramic #2 with a negative imprint of a plant offering (cola or palm nut?) (large arrow). Again, the profile and thickness of the entire ceramic, as well as its areas of weakness, appear and are measurable.

The study of the two ceramic vessels (“*canaris*”) #1 and #2 revealed a sedimentary infill consisting of the progressive degradation of the initial offerings (food for ancestors and gods). Within these offerings, traces of plant offerings (hypodense areas (330–450 Hounsfield units [HU]) whose morphology and sizes are compatible with peanuts, seeds of jujube, cola and/or palm nut: this highlights the limitations of radiological examination, which identifies phantoms and shapes without any certainty about their true botanical nature) and a 4.5 mm of thickness declivitous area of low density (200 to 400 HU), likely corresponding to fatty deposits from past palm oil libations, can still be identified (Fig. 2, Fig. 3).

**Fig. 4:**
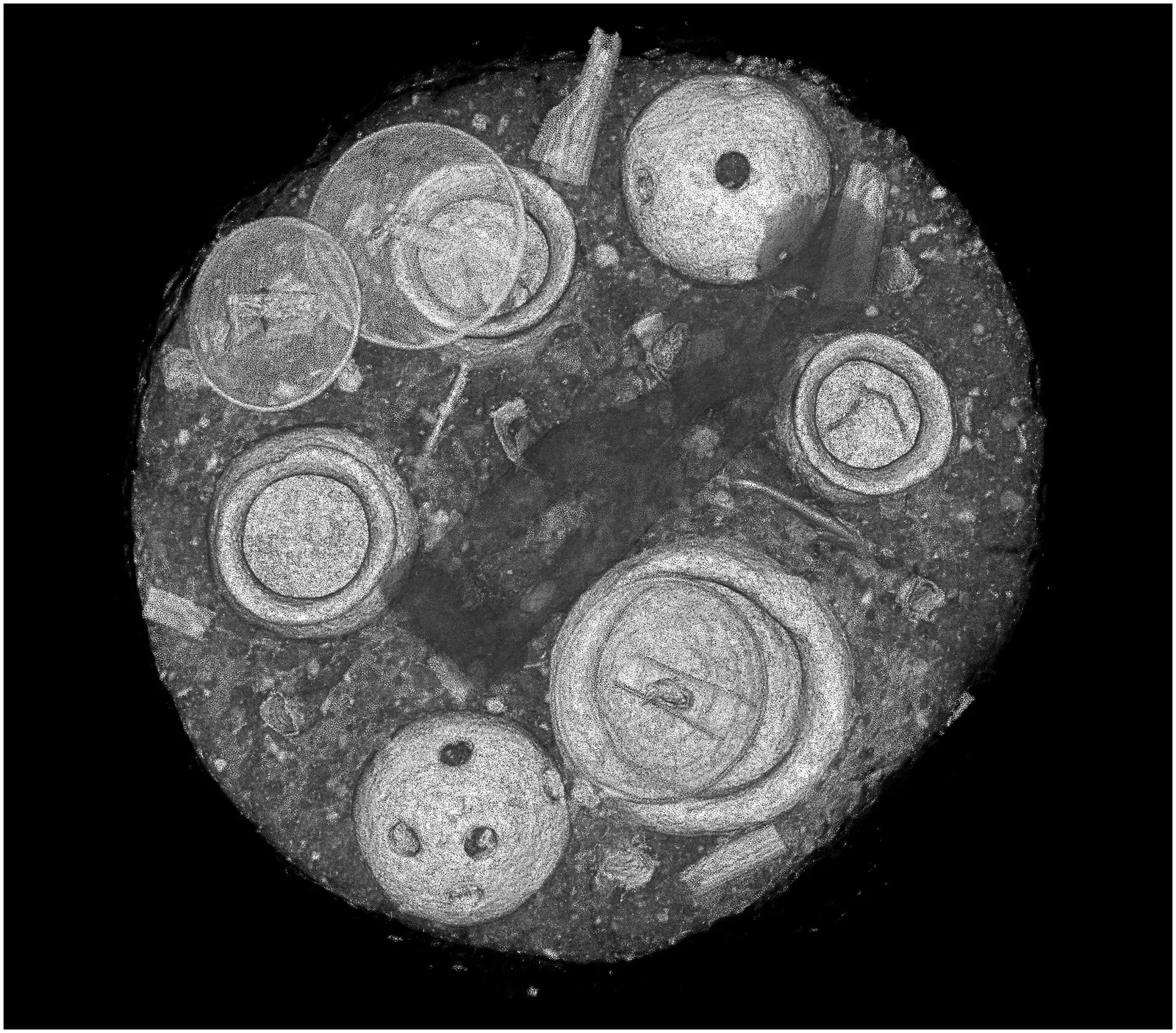
CT-scan examination of sacred Vodun objects (Benin): Horizontal view of the three-dimensional reconstruction in semi-transparency of the portable altar (#3) highlighting the internal structure of the objects stuck in the thick crust of sacrificial materials (in the center, in hypodensity (50 to 100 Hounsfield units), an anthropomorphic wooden statuette). Statement animals humans. This study does not involve humans nore animals, living or dead.

The study of the calabash (a portable altar dedicated to the judicial deity Heviosso, associated with social justice and celestial forces, particularly lightning) revealed its internal structure (Fig. 4): the complete morphology of the ceramic vessels (950 to 1050 HU) embedded in the layer of sacrificial materials, and votive offerings buried deep within (cowrie shells, 750 to 950 HU, originating from the coasts of East Africa, formerly used as currency during slave trade and now as offerings to deities).

## 4 Discussion

The ethical perspective of the Vodun community is essential in this scientific study. Therefore, several co-authors of this publication (CG, FH, VH and DA), as high-rank Vodun worshippers, had the opportunity to reflect on the appropriateness of such an examination, as well as to define the anthropological significance and precise status of these so-called "desacralized" objects. Specifically, an object from a Vodun ritual found in archaeological excavations (and thus "abandoned" on the ground for centuries, like offering ceramics) and an object collected from a priest (*bokonon*) market (commonly, but inaccurately, called a "fetish market") are considered "desacralized." This desacralization in no way diminishes their sacred and venerable character; it is merely a dormancy, a deactivation of the interaction between the deity or spirit embodied in the object and the practitioners. In this context, and after consulting with high-ranking practitioners of Beninese Vodun, according to some practical conditions and precautions, non-invasive and non-destructive act of scientific exploration may not be perceived as a lack of respect towards such objects and the supernatural entity (*vodun*) that still resides within it, even in a very subtle trace.

This example illustrates not only the technical utility of the CT-scan tool for the study of archaeological objects, but also its ethical justification since it allows us to "know without destroying", thus respecting as much as possible the ethical wishes of the communities of origin. But beyond individual case studies, medical CT imaging is progressively shaping a genuine field of “heritage radiology” situated at the crossroads of medical imaging, archaeometry, and conservation science. In museums and collections, CT-scan does not merely reveal hidden contents; it documents manufacturing techniques, repairs, inclusions, structural weaknesses, and previous restoration materials that are otherwise inaccessible without invasive sampling [6]. The radiological dataset becomes an archival layer in its own right, enabling longitudinal monitoring of object stability, virtual handling of fragile items, and the sharing of digital surrogates between institutions without transporting the original artifact. In this sense, the CT-scan functions not only as a diagnostic instrument but also as a preventive conservation tool, contributing to risk assessment before exhibition, transport, or climatic changes. Radiologists thus participate directly in heritage stewardship, extending their traditional clinical role into the domain of material biography and object life history.

An essential dimension of this study concerns the ethical framework within which the CT-scan examinations were conducted. As previously precised, these Vodun ritual objects are not considered inert artifacts by local communities and adepts, but rather sacred entities (even if of archaeological origin) embodying more or less important “quantities/qualities” of spiritual forces and ancestral presences (for objects recovered from archaeological excavations, their "dead" or "living" nature can be debated depending on the perspective of a heritage curator or a follower of the religion whose remains were unearthed). Consequently, any invasive intervention, opening, or sampling could be perceived as a symbolic mutilation or desacralization. The importance of the hands that will touch, place, and manipulate these objects is essential: while not risking desecration, corruption of the objects is theoretically possible, requiring subsequent purification rituals to return them to ritual use. Ideally, at least one adept should be present to guide the process, or at least handle the object themselves, for example, to transport it and place it on the moving CT-scan table.

For this reason, this study was developed in close dialogue with local stakeholders, including members of the Vodun community, cultural mediators, and authorities associated with the preservation of the royal heritage of Abomey. The choice of CT imaging specifically resulted from a shared ethical objective: to document and study the objects while trying to preserve at a maximum their material and symbolic integrity. This collaborative approach constitutes a major technical and ethical advance in the direction of respect local cultural values and ritual sensitivities during such scientific investigations. More broadly, the study highlights the importance of integrating community consultation, cultural sensitivity, and non-destructive methodologies into research protocols involving sacred or culturally sensitive heritage objects, especially in postcolonial contexts where questions of ownership, interpretation, and respect for traditional beliefs remain central, if not essential.

However, such a transfer of medical imaging technology to cultural heritage contexts also raises methodological challenges. Recent publications in this field of archaeological radiology highlights that differences in density between organic residues, mineral inclusions, corrosion products, and restoration materials can produce ambiguities that require interdisciplinary interpretation (and experience) combining radiological expertise with archaeological, chemical, and art-historical knowledge [7], [8], [9], [10], [11], [12], [13], [14], [15], [16], [17]. As for forensic imaging, standard clinical acquisition protocols are not always optimal for heterogeneous archaeological materials, prompting adaptations in voltage, filtration, and reconstruction algorithms to balance penetration power and contrast resolution [18]. Moreover, CT-scan data should be understood as part of a multianalytical strategy rather than a self-sufficient truth: it visualizes structure and density contrasts, not composition or symbolic meaning. In general, radiology therefore completes but does not replace traditional examination, laboratory analyses, and/or ethnographic context, reinforcing a model of non-destructive investigation that is both technologically advanced and epistemologically cautious.

## Declaration of competing interest

The authors declare that they have no known competing financial interests or personal relationships that could have appeared to influence the work reported in this paper.
